# Supplementary material for: Breast Cancer Chemotherapeutic Options: A General Overview on the Preclinical Validation of a Multi-Target Ruthenium(III) Complex Lodged in Nucleolipid Nanosystems
Source: Cells. 2020 Jun 5;9(6):1412. doi: 10.3390/cells9061412 (PMC7349411; doi:10.3390/cells9061412)
Supplement: Supplementary file 1 [file cells-09-01412-s001.pdf]

Statistical dataset relating to preclinical studies in vitro on Ru-based nanosystems

Throughout the study by cellular models, consideration was rewarded to the statistical analysis of the experimental data to determine the results impact and to ensure their reproducibility

The antiproliferative activity of nucleolipid nanosystems enclosing the anticancer ruthenium(III) complex AziRu was investigated by specific bioscreens in vitro on selected panels of human cancer cell lines through the estimation of a "cell survival index", resulting from the combination of a functional assay (MTT) for mitochondrial redox activity with TC20 automated cell count [78]. Results were expressed as percentage of untreated control cells and are reported as mean values ± SEM (n = 30) of five independent experiments (6 replicates for each in vitro treatment, including untreated controls) [57, 130, 140]. For each compound and nanoformulation under investigation, concentration-response curves were then elaborated. Similarly, the calculation of the concentration required to inhibit the net increase in the cell number and viability by 50% (IC_{50}) is based on plots of data (n = 6 for each experiment) and repeated five times (total n = 30). IC_{50} values were obtained starting from concentration response curves by nonlinear regression using a curve fitting program (Graph-Pad software Inc., San Diego, CA), and they were expressed as mean values ± SEM [57, 78, 129, 130, 139, 140, 141].

Sub-cellular bioaccumulation and localization of the AziRu complex following treatments were evaluated by inductively coupled plasma-mass spectrometry (ICP-MS) analysis, where results derive from the average ± SEM values of three independent experiments. Each experimental group was of 6 replicates, achieving a total of n = 18 for each treatment [78].

Data by immunostaining, confocal and fluorescent microscopy derive from the average ± SEM values of three independent experiments. Each experimental group was made of 6 independent replicates, with a total of n = 18 for each treatment in vitro [57, 78, 129, 130, 140].

Protein expression performed by WB analysis was ensured by densitometric analysis and plotted in line and/or bar graphs as percentage of controls, showing the average ± SEM values of four independent experiments, in turn consisting of 6 replicates (n = 24 for each treatment) [57, 78].

DNA fragmentation assay was performed by three independent experiments consisting of 6 replicates, for a total of n = 18 for each treatment in vitro [57, 140].

Analysis of apoptotic and autophagic cells was performed by flow cytometry (FACScan). For each sample, 2×10^4 events were acquired, and the study was carried out by triplicate determination on at least three separate experiments (n=9) [57].

All experimental data obtained during the preclinical studies have been subjected to statistical analysis by using Graph-Pad Prism (Graph-Pad software Inc., San Diego, CA). ANOVA test for multiple comparisons was performed followed by Bonferroni's test.
